# Recruited lung tissue does not resume normal mechanical properties

**DOI:** 10.1186/cc12044

**Published:** 2013-03-19

**Authors:** M Cressoni, C Chiurazzi, M Amini, D Febres, E Gallazzi, E Carlesso, P Cadringher, T Langer, D Chiumello, L Gattinoni

**Affiliations:** 1 Università degli Studi di Milano, Milan, Italy; 2 Fondazione IRCCS Ca' Granda - Ospedale Maggiore Policlinico, Milan, Italy

## Introduction

Positive end-expiratory pressure (PEEP) is commonly used in patients with ARDS to prevent end-expiratory collapse. The increase in gas volume and/or change in mechanical properties of the lung are used at the bedside to estimate lung recruitment and individualize PEEP selection.

## Methods

Retrospective analysis of patients who underwent whole-lung CT scan at 5 and 15 cmH_2_O PEEP. Every chest CT was divided both into 10 apex-base levels of equal height and into 10 sterno-vertebral levels, yielding 100 units per scanned lung (200 units per patient); each unit was classified according to its average CT number (<-100 not inflated, -100 to -500 poorly inflated, -500 to -900 well inflated and -900 overinflated). Lung regions defined as not inflated at PEEP 5 cmH_2_O and inflated (poorly, well or over) at PEEP 15 cmH_2_O were classified as recruited. Moreover, a surrogate specific lung compliance (CLsp) was determined on the expiratory limb of the pressure-volume curve (15 to 5 cmH_2_O PEEP at CT) and was defined as Δgas/cmH_2_O PEEP/gram of tissue.

## Results

We included in our study the CT scans of 89 patients (24 mild, 55 moderate and 10 severe ARDS, age 58 ± 17 years, BMI 25.6 ± 5.4, PaO_2_/FiO_2 _165 ± 66, PaCO_2 _42 ± 9, 60 discharged alive from ICU (67%)). At PEEP 5 cmH_2_O 39 ± 16% of the lung parenchyma was not inflated, 33 ± 13% poorly inflated, 28 ± 15% well inflated and 0 ± 1% overinflated. The median lung recruitability between 5 and 15 cmH_2_O PEEP was 6% (3 to 10%) of lung parenchyma (71 g (26 to 182 g)). Mean EELV at PEEP 5 cmH_2_O was 1,233 ± 709 while it increased to 1,784 ± 848 at PEEP 15 cmH_2_O (ΔEELV = 551 ± 407 ml). More than one-half (52% (31 to 75%)) of the increase in end-expiratory lung volume (EELV) was due to an increase in inflation of the already well-aerated tissue; 38% (23 to 43%) was due to the inflation of poorly aerated tissue, while only 4% (1 to 8%) to the recruitment of lung tissue. The CLsp of the well, poorly and recruited tissue was 0.07 (0.031 to 0.13), 0.03 (0.01 to 0.06) and 0.02 (0.01 to 0.04) ml/cmH_2_O/g, respectively (*P <*0.0001).

## Conclusion

Most of the PEEP-related EELV increase is due to an increased inflation of already well aerated lung tissue. In agreement with the baby lung theory [1], the well-aerated tissue showed near to normal mechanical properties.
